# Response to dietary tannin challenges in view of the browser/grazer dichotomy in an Ethiopian setting: Bonga sheep versus Kaffa goats

**DOI:** 10.1007/s11250-015-0931-3

**Published:** 2015-10-30

**Authors:** Kechero Yisehak, Yoseph Kibreab, Tolemariam Taye, Marta Ribeiro Alves Lourenço, Geert Paul Jules Janssens

**Affiliations:** Department of Animal Sciences, Arba Minch University, P.O. Box 21, Arba Minch, Ethiopia; Bonga Agricultural Research Centre, P. O. Box 309, Bonga, Ethiopia; Department of Animal Sciences, Jimma University, P.O. Box 307, Jimma, Ethiopia; Laboratory of Animal Nutrition, Faculty of Veterinary Medicine, Ghent University, Heidestraat 19, 9820 Merelbeke, Belgium

**Keywords:** Digestibility, Goat, Nutrition, Polyethylene glycol, Sheep, Tannin

## Abstract

It has been suggested that goats (typical browser) are better adapted to digest tannin-rich diets than sheep (typical grazer). To evaluate this, Bonga sheep and Kaffa goats were used in a 2 × 3 randomized crossover design with two species, three diets, and three periods (15-day adaptation + 7-day collection). The dietary treatments consisted of grass-based hay only (tannin-free diet = FT), a high-tannin diet (36 % *Albizia schimperiana* (AS) + 9 % *Ficus elastica* (FE) + 55 % FT (HT)), and HT + polyethylene glycol 6000 (PEG). Animals were individually fed at 50 g dry matter (DM)/kg body weight (BW) and had free access to clean drinking water and mineralized salt licks. Nutrient intake, apparent nutrient digestibility, nutrient conversion ratios, and live weight changes were determined. Condensed tannin concentrations in AS and FE were 110 and 191 g/kg DM, respectively. Both sheep and goats ate 47 % more of HT than FT, and dry matter intake further increased by 9 % when PEG was added, with clear difference in effect size between goats and sheep (*P* < 0.001). The effects of the tannin-rich diet and PEG addition were similarly positive for DM digestibility between sheep and goats, but crude protein (CP) digestibility was higher in HT + PEG-fed goats than in sheep fed the same diet. However, PEG addition induced a larger improvement in growth performance and feed efficiency ratio in sheep than in goat (*P* < 0.001). The addition of PEG as a tannin binder improved digestion and performance in both species, but with the highest effect size in sheep.

## Introduction

Despite the previous, several animal species including ruminants (*Bos indicus* cattle: Yisehak et al. 2011), and rhinoceros species (Clauss et al. 2005) and primates (*Papio hamadryas*: Shimada 2006) seem to tolerate (or even prefer) considerable amounts of tannins (<50 g condensed tannin (CT)/kg dry matter (DM)) in their diets. Nonetheless, tannin coping strategies vary among different classes of animals (Lamy et al. 2011; Yisehak et al. 2012).

Whatever the classification, there is a general consensus in considering that goats eat proportionally more browsing material than sheep. Goats usually have the capacity of adapting their ingestive behavior to food items available and select diet compounds in order to maintain the essential nutrients and tannin proportions relatively constant throughout the year (Kharrat and Bocquier 2010).

Under natural grazing and browsing conditions, animals are exposed to several tannin sources and concentrations. In order to understand the difference between browser and grazers in terms of feed efficiency and performance upon consuming tannin-rich diets, an experimental study was designed. Further, polyethylene glycol 6000 (PEG; a powerful tannin-binding agent; Makkar 2003) was added or not to the diet of sheep and goats, in order to assess to which extent this strategy can help browsers and grazers to overcome the anti-nutritional effects of tannins. The level of PEG used was 40 mg PEG/kg CT on DM basis. This level of PEG addition might be considered low compared to that in the literature, but if it would be successful, it could easily be accepted, available, and used by smallholder farmers (acceptable costs) and could further help in developing a more general feeding strategy for other sheep and goat breeds in the tropics. In order to evaluate if tropical goats better digest a tannin-rich diet than tropical sheep, and that the application of a PEG would have the largest effect in sheep, this study was conducted with dietary inclusion of leaves of the CT-rich trees (*Albizia schimperiana*, AS, and *Ficus elastica*, FE) in grass-based hay either with or without PEG on nutrient intake and digestibility, weight change, feed conversion, and protein efficiency ratio in Ethiopian Bonga sheep and Kaffa goats.

## Materials and methods

### Collection and preparation of experimental diets

The basal diet (hay) was harvested from a natural pasture site of Jimma University, Ethiopia (37° 039′ 57″ N, 37° 48′ 59″ E, and 1705 m above sea level). The plant composition of the natural pasture hay was assessed directly before harvest, and the biomass proportion was expressed on DM basis (Baars et al. 1997). The hay was composed of about 52.0 % *Poaceae*, 30.0 % *Asteraceae*, 17.5 % *Fabaceae*, and 0.50 % *Cyperaceae* and *Juncaceae*. The natural pasture was cut when major plants attained 50 % flowering stage. Cut hay was left to dry for 3 days (±90 % DM). The hay was stored in bales under shade until use as the basal diet.

*A. schimperiana* and *F. elastica* were selected because of their high crude protein (CP) content, superior fodder biomass, and wide distribution in the study region, and because they are commonly consumed by herbivores (Yisehak et al. 2010). Fresh leaves of *A. schimperiana* and *F. elastica* were hand-plucked from 30 randomly selected farm-grown trees. Leaves from the different trees were pooled together and taken to the small ruminant research facility of Jimma University College of Agriculture and Veterinary Medicine, within 40 min. After arrival, the fresh leaves were spread on a plastic sheet and left to dry for 3 days under shade (25 °C). After air drying (≥90 % DM), leaves were packed in polythene bags (50 kg DM per bag) and stored under shade until use as the test diet. This drying approach was chosen because oven drying of tannin-rich feeds, even at temperatures below 60 °C, is known to polymerize tannins and increase neutral detergent fiber (NDF), fiber-bound nitrogen, and lignin contents (Makkar 2003).

### Animals, experimental design, and feed management

Six Bonga rams (22.2 ± 2.90 kg) and six Kaffa bucks (23.1 ± 1.50 kg), of 1.20 year of age on average, were used. The sheep and goats were purchased from Seka livestock market in Jimma zone, southwest Ethiopia. Animals were allowed to adapt to the experimental conditions and basal diet for 1 month. Prior to experiment, the animals were dewormed against gastro-intestinal and external parasites using ivermectin and vaccinated against pneumonic Pasteurellosis and Blackleg. Pens were built in a well-ventilated shed with one side open to natural light and roofing to protect animals against sun and rain. Animals were randomly housed in individual holding pens (1.5 × 1.5 m^2^) with concrete floors on an open-air platform.

The experimental design was a randomized 2 × 3 crossover trial, with 21 days for each period, 2 weeks for adaptation, and 1 week for data collection. In the beginning and last day of each of the experimental periods, all animals were weighed individually following overnight fasting to avoid gut content variation. Diet allowance for the next period was recalculated according to body weight (BW).

During the whole experimental trial, animals had free access to clean drinking water and mineralized salt licks. Total diets were given to the animals at an estimated 50 g DM/kg BW daily (Osuji et al. 1993). Test diets were provided once daily at 8:00 a.m. prior to the provision of basal diet at 10:00 a.m. in a separate trough that individually opened for each pen. The offered and refused amounts of all feeds were recorded to estimate the actual voluntary feed intake for each treatment.

Sheep and goats received PEG at a rate of 40 mg PEG to 1.0 kg of AS + FE, after mixing it with water at a rate of 0.5 g PEG/ml. In general, the daily diet (basal + supplement) was balanced to provide 8.36 MJ/kg metabolizable energy and 70 g CP/kg on DM basis (NRC 2007). The treatment combinations are presented in Table 1. Polyethylene glycol 6000 (PEG), the most effective tannin-binding chemical agent (Makkar 2003), was supplied by Micron International Trading House PLC, Addis Ababa, Ethiopia.

**Table 1:** Lists of treatment combinations used in the experiment

Treatment	Composition
FT	Control (tannin-free hay mixture)
HT	*Albizia schimperiana* (36 %) + *Ficus elastica* (9 %) + hay (55 %)
HT + PEG	*Albizia schimperiana* (36 %) + *Ficus elastica* (9 %) + hay (55 %) + PEG

*FT* tannin-free (basal) diet, *HT* high-tannin-containing diet, *PEG* polyethylene glycol

### Sample collection

Total fecal collection was performed to assess total diet digestibility. For this, sheep and goats were fitted with fecal collection bags using harnesses. Animals were allowed to adjust to the fecal collection bags 3 days before true collection. Feces were quantitatively collected on a daily basis from each animal and weighed, and 10 % was sub-sampled, pooled on animal basis per period, and frozen (−20 °C) until laboratory analysis. Feed leftovers were removed daily at 08:00 a.m. and weighed. During the collection period, samples of test and basal diets and refusals were collected, composited by animal per period, ground (1-mm screen), and kept frozen (−20 °C) until laboratory analysis.

### Chemical analysis of feed and feces

Samples of feedstuffs and feces were analyzed for DM, CP, total ash, crude fiber (CF), and ether extract (EE) contents (AOAC 1995). Neutral detergent fiber (NDF) was determined following Van Soest et al. (1991). Acid detergent fiber (ADF) and acid detergent lignin (ADL) contents were determined according to AOAC (1995). Determination of total extractable CT was based on oxidative depolymerization of CTs in butanol-HCl reagent using 2 % ferric ammonium sulfate in 2 N HCl catalyst (Porter et al. 1986). For chemical analysis (excluding N), the feces samples were oven-dried at 105 °C for 24 h. Non-oven-dried but well-mixed feces were directly used for N analyses. All chemical analyses were carried out in duplicate.

Metabolizable energy intake (MEI; kJ ME/kg BW^0.75^) was estimated according to Luo et al. (2004), MEI = 533 + (43.2 × ADG (g/kg BW^0.75^)). Metabolizable energy (ME; MJ/kg DM) contents of total diets were predicted from the equations of Abate and Meyer (1997) as ME = 5.34 − 0.1365CF + 0.6926NFE − 0.0152NFE^2^ + 0.0001NFE^3^, where NFE is nitrogen-free extract. Protein efficiency ratio (PER; McDonald et al. 2010) was determined as PER = % Protein in diet × Weight of diet consumed.

### Statistical analyses

Analysis of variance was carried out following 2 × 3 factorial arrangements according to the repeated measures design using mixed model procedures (PROC MIXED) of SAS 2013 version 9.4. Tukey test procedure was used to obtain confidence intervals for all pairwise differences between means. Mean differences were considered significant at *P* ≤ 0.05. The appropriate statistical model is indicated below:

$$ {Y}_{\mathrm{i}\mathrm{jk}}=\mu +{A}_{\mathrm{i} + }{B}_{\mathrm{j}}+{C}_{\mathrm{k}}+A{C}_{\mathrm{i}\mathrm{k}}+{\varSigma}_{\mathrm{i}\mathrm{jk}} $$

where *Y*_ijk_ = the response due to the animal *i*, in period *j*, treatment *k*, and interaction effects; *μ* = the overall mean effect; *A*_i_ = the fixed effect of the *i*th sheep or goat (subject; *i* = 1, 2, 3… 6); *B*_j_ = the random effect of the *j*th collection period (*j* = 1, 2, 3); *C*_k_ = the fixed effect of the *k*th treatment (*k* = 1, 2, 3); *AC*_ik_ = the interaction effect between species *i* and treatment *k*; and Σ_ijk_ = the random error

## Results

The chemical compositions of feed ingredients used in this trial are presented in Table 2. Despite their high contents of tannins (>100 g CT/kg DM), the CP content of *A. schimperiana* and *F. elastica* leaves was 459 and 313 % higher than that of the basal diet (hay).

**Table 2:** The chemical composition (g/kg DM) and metabolizable energy (MJ ME/kg DM) of the feedstuffs used in the study

Diet sources	DM	Ash	OM	EE	CP	NDF	ADF	ADL	CT	ME
Hay, basal diet	914	117	883	39	63	653	511	129	–	9.74
*A. schimperiana*	910	77	923	31	289	417	309	110	110	8.50
*F. elastica*	906	111	889	29	197	445	314	103	191	9.15

*DM* dry matter, *OM* organic matter, *EE* ether extract, *CP* crude protein, *NDF* neutral detergent fiber, *ADF* acid detergent fiber, *ADL* acid detergent lignin, *CT* condensed tannin, *ME* metabolizable energy

Average daily intakes of feed nutrients are presented in Table 3. The highest dry matter intake (DMI) (*P* < 0.001) was recorded for lambs fed HT + PEG (hay, 55 % + *A. shimperiana*, 39 % + *F. elastica*, 9 % + PEG) compared to FT (control diet). Similarly, the highest DMI (*P* < 0.001) in goats was also recorded for HT + PEG compared with FT. The DMI of goats fed HT was found to be significantly higher than that of sheep fed the same diet (*P* < 0.001). In the present trial, feeding grass-based hay + *A. shimperiana* and *F. elastica* with PEG significantly improved DMI of sheep and goats compared with that of non-PEG groups (*P* < 0.001). Although goats had the highest DMI across the treatments groups, the effect size of PEG inclusion was higher in sheep (*P* < 0.001).

**Table 3:** Least squares means for daily nutrient (g/kg DM /d) and energy intake (MJ ME/kg DM) in sheep and goats fed hay with or without leaves of tannin-rich trees and with or without polyethylene glycol

Nutrients	Species	Treatment, mean				*P* value		
		Basal	HT	HT + PEG	SEM	L	T	L × T
DM	S	625^b^	916^b^	989^b^	0.392	<0.001	<0.001	<0.001
	G	631^a^	924^a^	993^a^				
CP	S	62^b^	91^b^	176^b^	0.481	<0.001	<0.001	<0.001
	G	69^a^	98^a^	185^a^				
EE	S	11^b^	18^b^	24^b^	0.478	<0.01	<0.01	<0.01
	G	14^a^	22^a^	27^a^				
OM	S	583^b^	731^b^	812^b^	0.425	<0.001	<0.001	<0.001
	G	586^a^	735^a^	817^a^				
ADF	S	244^b^	359^b^	438^b^	0.433	<0.001	<0.001	<0.001
	G	263^a^	380^a^	471^a^				
NDF	S	260^b^	370^b^	460^b^	0.232	<0.001	<0.001	<0.001
	G	280^a^	397^a^	490^a^				
ME	S	621^b^	876^b^	949^b^	0.326	<0.001	<0.001	<0.001
	G	673^a^	885^a^	957^a^				

Means with different superscripts in the same column are significantly different (*P* < 0.05)

*DM* dry matter, *CP* crude protein, *EE* ether extract, *OM* organic matter, *ADF* acid detergent fiber, *NDF* neutral detergent fiber, *ME* metabolizable energy, *SEM* standard error of mean, *S* sheep, *G* goats, *L* species, *T* treatment, *L* × *T* interaction effect of species and treatment

Apparent digestibility coefficients of nutrients in each treatment are presented in Table 4. In sheep, apparent dry matter digestibility (DMD) was higher for HT + PEG compared with that for the other treatment groups (*P* < 0.001). Similarly, in goats, the DMD for HT + PEG was also found to be the highest value compared to that for the other dietary treatments (*P* < 0.001). Goats showed superior digestion capability of DM than sheep fed the same diet, particularly HT + PEG, and the lowest values were recorded for FT (*P* < 0.001). Like for DMD, sheep had the highest organic matter digestibility (OMD; 67 %; *P* < 0.001) which were fed HT + PEG compared to FT or HT. Goats fed HT + PEG showed the same trend, i.e., higher OMD (69 %) was recorded with HT + PEG compared to that in other treatments (*P* < 0.001). The crude protein digestibility (CPD) was also superior (*P* < 0.001) for both animal species after feeding *A. shimperiana* and *F. elastica* with PEG than that for browse species without PEG or in FT. Goats had also higher CPD compared to sheep in all treatment groups (*P* < 0.001). The interaction effects between fixed and random factors were considerable for CPD for all treatment groups. Digestibility of ADF and NDF varied notably among animal species and treatments, where the highest fiber digestibility values were recorded for goats (*P* < 0.001). When fed PEG, sheep performed higher neutral detergent fiber digestibility (NDFD) (56 %) compared to FT which showed the lowest NDFD (46 %) (*P* < 0.001). Goats also performed better NDFD (63 %, *P* < 0.001) when fed with PEG compared to HT (non-PEG treatment, 53 % NDFD) and FT (control diet, 48 %), meaning that PEG treatment of high-tannin diet showed 15 % improvement in NDF digestibility and HT groups digested only 5 % more NDF compared to FT.

**Table 4:** Least squares means for apparent digestibility of nutrients (%) compared between sheep and goats fed hay with or without mixes of tannin-rich tree leaves with or without PEG

Nutrients	Species (L)	Treatment, %				*P* value		
		Basal	HT	HT + PEG	SEM	L	T	L × T
DM	S	50^b^	59^b^	66^b^				
	G	59^a^	68^a^	77^a^	0.114	<0.001	<0.001	<0.001
CP	S	42^b^	53^b^	67^b^				
	G	48^a^	65^a^	72^a^	0.091	<0.001	<0.001	<0.001
EE	S	48^b^	53^b^	60^b^				
	G	51^a^	58^a^	64^a^	0.220	<0.001	<0.001	<0.001
OM	S	53^b^	61^b^	67^b^				
	G	54^a^	63^a^	69^a^	0.016	<0.001	<0.001	<0.001
NDF	S	46^b^	50^b^	56^b^				
	G	48^a^	53^a^	63^a^	0.142	<0.001	<0.001	<0.001
ADF	S	37^b^	40^b^	45^b^				
	G	41^a^	45^a^	51^a^	0.055	<0.001	<0.001	<0.001

Means with different superscripts in the same column are significantly different (*P* < 0.05)

*DM* dry matter, *CP* crude protein, *EE* ether extract, *OM* organic matter, *ADF* acid detergent fiber, *NDF* neutral detergent fiber, *ME* metabolizable energy, *SEM* standard error of mean, *S* sheep, *G* goats, *L* species, *T* treatment, *L* × *T* interaction effect of species and treatment

Daily weight changes as shown in Table 5 were also highest in goats compared to those in sheep for all dietary treatments (*P* < 0.001). The PEG inclusion with feedstuffs highly improved weight gain and feed conversion ratio both in sheep and goats, yet the values were higher for goats (*P* < 0.001). Although goats had better use of high tannins in comparison to sheep, PEG inclusion further improved their feed conversion and protein efficiency ratios considerably (*P* < 0.001).

**Table 5:** Least squares means for ADG (g/day), FCR (g DMI/g ADG), and PER (g ADG/g CP) compared between sheep and goats fed hay with or without leaves of tannin-rich trees with or without PEG

Parameters	Species (L)	Treatment, mean					P-value	
		Basal	HT	HT + PEG	SEM	L	T	L × T
ADG	S	−2.5^b^	16^b^	37^b^	0.107	<0.001	<0.001	<0.001
	G	−2.0^a^	22^a^	41^a^				
FCR	S	−284^b^	57^b^	29^b^	0.020	<0.001	<0.001	<0.001
	G	−316^a^	49^a^	26^a^				
PER	S	−0.035^b^	0.16^b^	0.21^b^	0.07	<0.001	<0.001	<0.001
	G	−0.022^a^	0.19^a^	0.29^a^				

Means with different superscripts in the same column are significantly different (*P* < 0.05) science

*ADG* average daily weight gain, *FCR* feed conversion ratio (DM intake/ADG), *PER* protein efficiency ratio, *SEM* standard error of means, *S* sheep, *G* goats, *L* species treatment, *L × T* species-treatment interaction

## Discussion

The recommendation of 60–80 g CP/kg DM requirements for ammonia synthesis for optimum microbial activity in ruminants (NRC 2007) can be adequately met by the tannin-rich test diets. The intake of roughage is limited when their CP content is less than 100 g/kg DM (McDonald et al. 2010). The CP content of *A. schimperiana* or *F. elastica* also overqualifies these recommendations. Even supposing test diets had high concentration of CT (110–190 g/kg DM), the dietary inclusion of PEG could alleviate the inhibitory effects of CTs against feed use efficiency. At higher levels (>50 g CT/kg DM), tannins become highly detrimental (Makkar 2003) as they reduce digestibility of nutrients in the rumen by inhibiting the activity of bacteria and anaerobic fungi; high levels also lead to reduced intake. Brooker et al. (1999) also reported that ruminants consuming tannin-rich diets usually develop a negative nitrogen and energy balance and lose weight and body condition unless supplemented with non-protein nitrogen, carbohydrate, and minerals.

The larger improvement of DMI in HT after PEG inclusion could be connected with the selective tannin binding power of PEG. PEG has a higher affinity to tannins than do proteins (Hagerman and Butler 2010). Likewise, the improved intake of all other nutrients measured in the present study could be associated the tannin complexing competence of PEG. Despite the differences in the magnitude of nutrient consumption in goats and in sheep, the superior improvement of nutrient intake in both animal species after PEG addition can be a good scientific evidence for improvement of sheep and goat nutrition under smallholder farming settings in harsh environments through feeding new tannin-rich feedstuffs. The higher NDF intake in goats with or without PEG compared to sheep might be linked with the better CP utilization ability of goats on tannin-rich diets compared with that of sheep. The proficient CP utilization in goats can stimulate proliferation of fiber-degrading bacteria in the digestive tract of goats. It suggests that goats are more efficient in the digestion of fiber and the utilization of poor roughages than sheep (Kijas et al. 2012). In general, the improved capacity of goats consuming high-tannin browses and detoxifying the tannins compared to sheep under comparable conditions might be associated with the evolutionary adaptation of goat breeds to tannin-rich browses in tropical environments. It is assumed to be achieved through secretion of special tannin binding proteins (Agrawal et al. 2014).

The improvement of DM digestibility in goats and sheep against the high-tannin diets (CT >100 g/kg DM) might be associated with their adaptation to tannins. Several authors reported that tannins challenge feed digestion of ruminants at concentrations over 50 g CT/kg DM; yet, in the present study, it has been clearly observed that Bonga sheep and Kaffa goat breeds consumed tannin-rich diets with modest limitations by 45–60 % improvement of DM digestion. Dietary inclusion of high-tannin feedstuffs at rate of 45 % of total ration could be considered acceptable allowance for sheep and goat feeding. The higher DM digestion coefficients of goats over sheep for all treatment combinations might be associated with goat’s digestive physiology which appears to be associated with lower retention of ingested feeds (Lamy et al. 2011). Supplementation of PEG could also lead to efficient utilization of proteins and other essential nutrients in the goat’s rumen rather than escaping as bypass protein because of tannin binding. The tannin adapting physiological features in goats might be linked to a large absorptive area of their rumen epithelium, and a capacity to change the volume of the foregut rapidly in response to environmental changes.

The lower CPD both in sheep and in goats for non-PEG treatments might be associated with protein-binding effects of tannins. It has been reported that zebu cattle fed with dried leaves of several tanniferous trees and supplemented with PEG showed an increase of in vivo CP digestibility (Yisehak et al. 2011, 2014). Several authors have reported a reduction in protein digestibility in ruminants fed diets containing high levels of CTs. In addition to complexion with dietary proteins, CTs combine with and hinder digestibility of cellulose, hemicelluloses, and pectin either by preventing microbial digestion or by directly inhibiting cellulolytic microorganisms. Further, CTs were reported to form combination with proteins in the rumen rendering them unavailable for digestion and consequently increase their output in feces (Robins and Brooker 2005).

A better fiber digestibility (ADF, NDF) in goats over that in sheep by the side of high-tannin load reflects a better fiber-utilizing capability of goats. This capacity could be also attributed to their longer retention time of digesta in the rumen. Although goats are considered as opportunistic feeders with a very flexible foraging behavior, they are the most appropriate animals to utilize the high-fiber, low-nitrogen forage produced on shrub lands and woodlands (Do Thi Thanh Van 2006).

## Conclusion

Supplementing sheep and goats fed natural pasture hay with 45 % of high-tannin diet and PEG (40 mg PEG to 1 kg of high tannin feed) substantially improved the nutrient intake, digestibility, and growth performance. In comparison of sheep and goat breeds evolved in tannin-rich environments when fed tannin-rich diets, goats gained better than sheep for feed use and weight change. Strategies that reduce the dietary tannin load, such as PEG supplementation, therefore have a considerably better impact on overall zootechnical performance in sheep than in goats in tropical conditions.
